# MMP-responsive *in situ* forming hydrogel loaded with doxorubicin-encapsulated biodegradable micelles for local chemotherapy of oral squamous cell carcinoma†

**DOI:** 10.1039/c9ra04343h

**Published:** 2019-10-02

**Authors:** Wei Li, Cheng Tao, Jiexin Wang, Yuan Le, Jianjun Zhang

**Affiliations:** State Key Laboratory of Organic-Inorganic Composites, College of Chemical Engineering, Beijing University of Chemical Technology Beijing 100029 PR China zhangjj@mail.buct.edu.cn wangjx@mail.buct.edu.cn; Beijing Advanced Innovation Center for Soft Matter Science and Engineering, Beijing University of Chemical Technology Beijing 100029 PR China; Research Center of the Ministry of Education for High Gravity Engineering and Technology, Beijing University of Chemical Technology Beijing 100029 PR China

## Abstract

The complex construction within the oral cavity causes incomplete surgical resection of oral squamous cell carcinoma (OSCC) that may enhance the risk of recurrence and metastasis in the treatment. *In situ* forming injectable hydrogels with minimally invasive procedures, encapsulation stability and stimuli-responsive degradation have emerged as promising carriers for local drug delivery. In this study, doxorubicin (DOX) was first encapsulated in biodegradable poly(d,l-lactide)-poly(ethylene glycol)-poly(d,l-lactide) (PDLLA-PEG-PDLLA) micelles and then loaded into an *in situ* injectable hyaluronic acid (HA) hydrogel, which was cross-linked by a matrix metalloproteinase-2 (MMP-2)-responsive peptide (GCRDGPQGIWGQDRCG) through a Michael addition reaction. *In vitro* studies demonstrated that the HA hydrogel had a sensitive MMP-2-responsive drug release profile. Investigations including MTT, live-dead, apoptosis, and wound healing assays illustrated that DOX micelle-loaded HA hydrogels exhibited outstanding cytotoxicity against squamous carcinoma cells (SCC-15). Furthermore, by *in vivo* studies, we also proved that HA hydrogels degraded faster in the tumor site than in normal tissue, which led to a local sustained release of DOX-loaded micelles and tumor growth inhibition of oral squamous cell carcinoma (OSCC) without any damage to the organs. Therefore, this work provides a remarkable drug delivery platform for local chemotherapy and other applications.

## Abstract 2

The complex construction within the oral cavity causes incomplete surgical resection of oral squamous cell carcinoma (OSCC) that may enhance the risk of recurrence and metastasis in the treatment.

## Graphical abstract

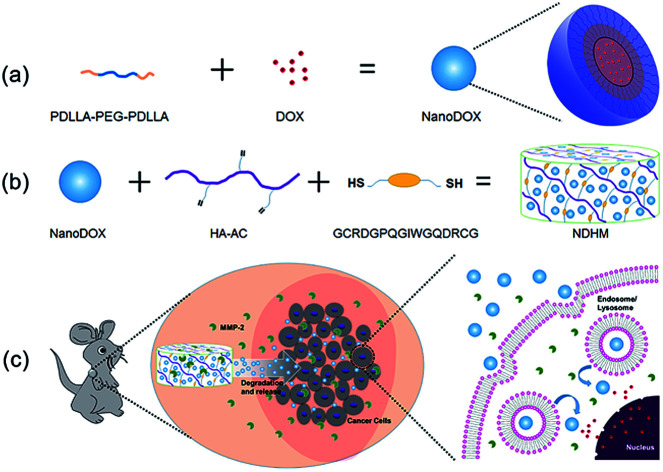

## 1 Introduction

Oral squamous cell carcinoma (OSCC) is the most common malignancy of the head and neck,^1^ and it is the sixth leading cancer by incidence worldwide with approximately 600 000 new cases reported annually.^2–4^ Although unceasing progress in clinical cancer treatments has been made in recent years, the 5 year survival rate of OSCC hovers at approximately 50%.^4,5^ Anatomically, there are many anatomic subsites in the oral cavity, including the labial mucosa, buccal mucosa, floor of the mouth, alveolar ridge and gingiva, anterior two-thirds of the tongue (anterior to the circumvallate papillae), hard palate, and retromolar trigone.^1^ The complex construction within the oral cavity causes incomplete surgical resection that leads to an unsatisfactory therapeutic effect and enhances the risk of recurrence in the treatment of OSCC. Therefore, the development of local drug delivery systems with long-term sustained, on-demand or smart-responsive drug release behavior may offer distinct advantages for chemotherapy and postoperative adjuvant therapy of OSCC by increasing the local drug bioavailability and reducing the adverse effects of drugs.

*In situ* forming injectable hydrogels with minimally invasive procedures, high biocompatibility and desirable bioactivity have been widely developed for drug delivery and tissue engineering applications.^6–10^ The encapsulation of the therapeutic drugs into the hydrogel depots for the treatment of local disease has many attractive advantages.^11–13^ Thus, by molecular design and manipulation of the preparation parameters, such as the content and cross-linking density, the drug release behavior from hydrogel depots can be significantly controlled for improving the drug bioavailability, action time and unanticipated adverse effects. Furthermore, a variety of stimuli-responsive factors such as acidity,^14^ light,^15^ magnetism,^16^ temperature,^17^ and enzymes,^18^ can be utilized to design and fabricate smart hydrogel materials for local sustained drug release aimed at achieving the optimal therapeutic efficacy while reducing the side-effects of drugs.

Among those stimuli-responsive factors, specific enzymes have attracted enormous attention due to their striking and robust characteristics.^19^ Therefore, enzyme-responsive drug release systems have been extensively explored for the treatment of various diseases, especially for cancer.^20^ Matrix metalloproteinase-2 (MMP-2) is overexpressed in many types of cancer including OSCC, and it has been reported that MMP-2 plays a key role in cancer invasion, progression, recurrence and metastasis.^21^ The peptide GPQGIWGQ is one of its known substrates, which can be selectively cleaved by MMP-2.^22–26^ Thus incorporating GPQGIWGQ into a cross-linked structure may endow hydrogels with MMP-2-responsive degradability that is suitable for fabricating local stimuli-responsive drug release systems for tumor treatment and the prevention of recurrence.^27–31^

For local hydrogel drug delivery systems, only when the drugs disperse homogeneously in a hydrogel matrix can drug release follow a constant rate through passive diffusion or hydrogel degradation. However, most chemotherapeutic drugs are hydrophobic molecules, and they cannot stably disperse in aqueous solution or hydrogel precursor solution.^32^ One of the best methods to deal with this problem is preparing drug-encapsulated amphiphilic polymer micelles by using the solvent-antisolvent method.^33–35^ To date, amphiphilic block copolymers composed of the hydrophilic chain of poly(ethylene glycol) (PEG) and the hydrophobic chain of polyesters, such as poly(l-lactide) (PLLA), poly(d,l-lactide) (PDLLA), poly(lactic acid-*co*-glycolic acid) (PLGA), and poly(caprolactone) (PCL), have been widely developed for water-insoluble drug delivery due to their outstanding ability for self-assembly, stability in the aqueous phase, biocompatibility, and biodegradability,^36–41^ and some of the drug formulations containing such polymers have already been approved by the Food and Drug Administration (FDA).^33,42^

**Scheme 1:**
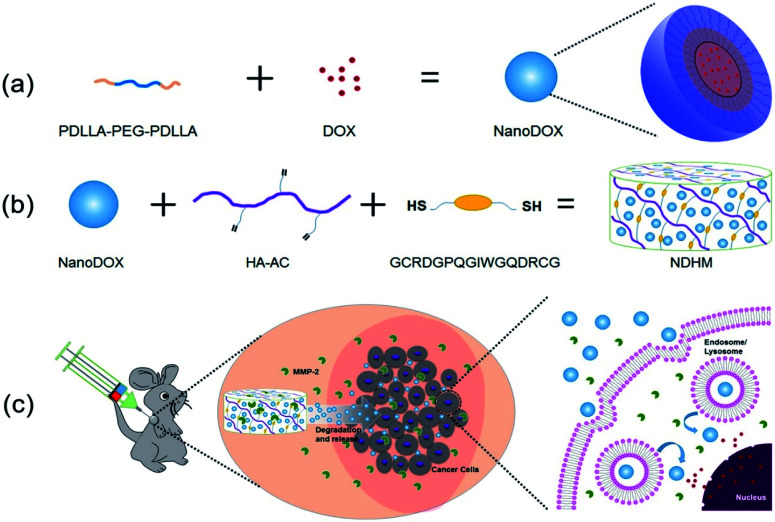
Schematic illustration showing (a) the preparation process of NanoDOX and (b) the preparation process of NDHM. (c) NDHM is injected and formed in the tumor site of the mouse by a disposal-connected mixing system, and then, NDHM can be degraded by MMP-2 in the tumor microenvironment, leading to NanoDOX spreading in the tumor tissue and further entering into tumor cells. Finally, DOX released from NanoDOX will enter into the nucleus and intercalate on DNA to induce programmed cell death.

In this study, we developed a MMP-2-responsive *in situ* forming injectable hyaluronic acid (HA) hydrogel, which was used as the depot of doxorubicin (DOX)-encapsulated biodegradable micelles for the local chemotherapy of OSCC. As shown in Scheme 1, we first synthesized an amphipathic PDLLA-PEG-PDLLA triblock copolymer, and DOX was loaded into the PDLLA-PEG-PDLLA to form DOX/polymer micelles (named as NanoDOX) by the solvent-antisolvent method. Then, NanoDOX was mixed with the hydrogel precursor solution of acrylated-HA (HA-AC),^43^ and this mixed solution was further cross-linked by a peptide (GCRDGPQGIWGQDRCG), which contained a MMP-2 cleavable sequence (GPQGIWGQ) and two cysteine (C) residues, through a Michael addition reaction to form a hydrogel drug depot. For the *in vivo* experiment, the mixed solution of HA-AC and NanoDOX was directly injected with peptide solution at the tumor site through a disposal-connected mixing system, and the hydrogel drug depot was formed *in situ*. Furthermore, by the up-regulation of MMP-2 expression in OSCC, the hydrogel depot was continuously degraded to release the loaded NanoDOX. Finally, the free NanoDOX was spread and entered into the cancer cells to play the role of a chemotherapeutic agent. Moreover, *in vitro* studies demonstrated that this NanoDOX-loaded HA-MMP hydrogel (NDHM) exhibited a sensitive MMP-2-responsive drug release profile and significant cytotoxicity against squamous cells (SCC-15). The *in vivo* investigations proved that hydrogel depot can effectively degrade and release the loaded NanoDOX in the tumor site, and it strongly inhibited the tumor growth of OSCC as well as reduced the side effects of drugs.

## 2 Experimental section

### 2.1 Materials

Doxorubicin hydrochloride (DOX·HCl) was purchased from the Zhongshuo Pharmaceutical Technology Development Co., Ltd, Beijing, China. Polyethylene glycol (PEG, *M*_n_ = 10 kDa) was purchased from Aladdin. d,l-Lactide (d,l-LA) was purchased from the Daigang Biomaterial Co., Ltd, Jinan, China. Sodium hyaluronan (HA, *M*_n_ = 60 kDa) was purchased from the Bloomage Freda Biopharm Co., Ltd, Jinan, China. *N*-Acryloxysuccinimide (NAS) was purchased from J&K Scientific Ltd, Beijing, China. MMP-sensitive peptides (GCRDGPQGIWGQDRCG) were synthesized by Scilight Biotechnology (Beijing, China). Active human recombinant MMP-2 was purchased from EMD Chemicals, Germany. SCC-15 cells were obtained from the American Type Culture Collection (ATCC, VA, USA). All other chemicals were purchased from the Chemical Reagent Company (Beijing, China) unless otherwise noted.

### 2.2 Characterization

^1^H NMR spectra were recorded on a Bruker AV-400 NMR spectrometer at room temperature. The chemical shift at 4.7 ppm was referred to as the solvent peak of D_2_O. Gel permeation chromatography (GPC, Waters 1515, US) was used to determine the macromolecular weight and macromolecular weight distribution of the prepared copolymers. Tetrahydrofuran (THF) was used as the mobile phase at a flow rate of 0.6 mL min^−1^ at 40 °C. The system was calibrated using monodispersed polystyrene (PS) standards. The size and zeta potential of NanoDOX in phosphate-buffered saline (PBS, 10 mM, pH = 7.4) buffer was determined by dynamic light scattering (DLS) using a Malvern Zeta Sizer Nano Instrument (ZS90, Malvern, UK). The measurements were carried out at room temperature. The morphology of NanoDOX was investigated by transmission electron microscopy (TEM, HT7700, Hitachi, Japan) operating at 300 kV. A TEM sample was prepared by directly depositing a drop of the sample solution onto a 200 mesh carbon-coated copper grid and leaving it to dry at room temperature for one day. NDHM was put in the refrigerator at −20 °C overnight, and then, it was broken in the middle and freeze-dried. Scanning electron microscopy (SEM, JSM-6701, JEOL, Japan) was used to observe the microstructure and distribution of NanoDOX in the hydrogels. Dynamic mechanical thermal analysis (DMTA V, Rheometric Scientific, USA) was used to analyze the loss modulus (*E*′) and storage modulus (*E*′′) of NDHM, and the tests were run from 0.1 Hz to 2 Hz.

### 2.3 Synthesis of PDLLA-PEG-PDLLA triblock copolymers

PEG (10 kDa) and d,l-lactide at a weight ratio of 3 : 1 were added to a round-bottom flask, which was put in an oil bath with stirring at 110 °C and further kept in a vacuum oven for 6 h to remove moisture from the reactant. After that, a certain amount of tin 2-ethylhexanoate was added to the flask under argon, and then, the mixture was reacted at 135 °C for 24 h. The product was collected, dissolved in dichloromethane and then dropwise added into cooled *n*-pentane with stirring. The solid was isolated, and the above-mentioned sedimentation step was repeated once more. Finally, PDLLA-PEG-PDLLA triblock copolymers were obtained after vacuum drying and then used without further purification.

### 2.4 Synthesis of HA-AC

HA-AC was prepared using a two-step synthesis. First, 2.0 g of hyaluronic acid (60 kDa) and 25 g of adipic dihydrazide (ADH) were dissolved in 400 mL of deionized water, and 2.5 g of 1-ethyl-3-[3-dimethylaminopropyl]carbodiimide hydrochloride (EDC) was added at a pH of 4.75. After the overnight reaction, the intermediate, HA-ADH was purified through dialysis with a molar weight cut-off (MWCO) of 8000 Da in deionized water for 7 d. Then, it was freeze-dried and stored at −20 °C until use. The modification of the carboxyl groups with ADH was 30.5% based on the trinitrobenzene sulfonic acid assay (TNBSA, Pierce, Rockford, Illinois). Next, 2 g of HA-ADH was completely dissolved in 400 mL of 2-[4-(2-hydroxyethyl)piperazin-1-yl]ethanesulfonic acid (HEPES) buffer (pH = 7.2), and 10 mL of DMSO containing 1.4 g of *N*-acryloxysuccinimide (NAS) was added dropwise to the buffer. After overnight reaction, the product was purified through dialysis with a MWCO of 8000 Da in deionized water for 7 d and then freeze-dried and stored at −20 °C until use.

### 2.5 Preparation of NanoDOX

DOX·HCl was dissolved in DMSO, and triethylamine (TEA) was added into the solution to neutralize the HCl of DOX·HCl. Then, the solution of DOX was added into the PBS buffer (150 mM, pH = 7.4) containing PDLLA-PEG-PDLLA at a concentration of 20 mg mL^−1^ with vigorous stirring for 1 h at room temperature. The suspension was dialyzed against deionized water using a dialysis bag with a MWCO of 3500 Da to remove unloaded DOX and DMSO, and then, it was freeze-dried to obtain NanoDOX powder.

The calibration curve of DOX in DMSO was determined at 488 nm by an UV-vis spectrophotometer (Varian Cary 50, CA, USA). Then, NanoDOX was dissolved in DMSO to measure the drug-loading capacity (DLC) and the encapsulation efficiency (EE) of NanoDOX. It was calculated according the following formula:
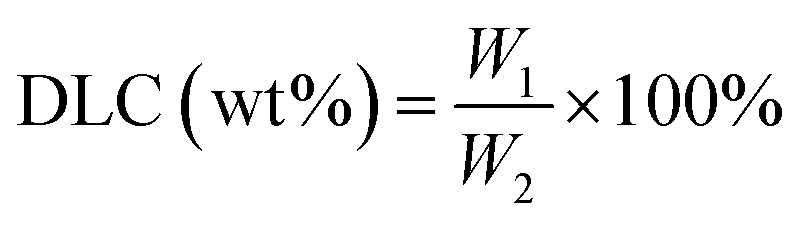
where *W*_1_ is the weight of DOX loaded into NanoDOX and *W*_2_ is the weight of NanoDOX.
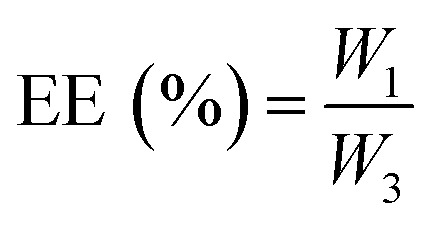
where *W*_3_ is the initial weight of the drug.

The stability test of NanoDOX in PBS was studied by DLS. The NanoDOX were dispersed in PBS (pH 7.4, 150 mM) as final concentration of 0.2 mg mL^−1^, and the detection period was 30 d.

### 2.6 *In vitro* DOX release from NanoDOX

The *in vitro* drug release from NanoDOX was studied in fresh PBS (150 mM, pH = 7.4). NanoDOX in 2 mL of PBS was put into a dialysis bag with a MWCO of 3500 Da. The dialysis bags were then immersed in 5 mL of buffer in sealed glass bottles. At each specified time point, 2 mL of the release medium was withdrawn from the bottles and replaced with 2 mL of fresh PBS (150 mM, pH = 7.4). The released DOX concentrations in the 2 mL solutions were measured by an UV-vis spectrophotometer at 500 nm. The cumulative drug release was calculated from the following formula:
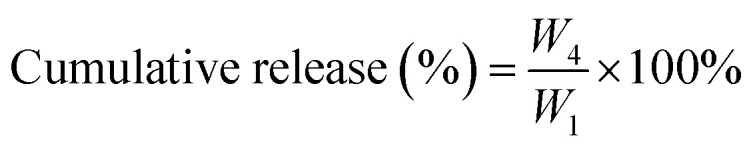
where *W*_4_ is the weight of DOX cumulatively released from NanoDOX at a specified time point.

### 2.7 Preparation of NDHM

The lyophilized powder of NanoDOX was dispersed in triethanolamine (TEOA, 300 mM, pH = 8.0) buffer under ultrasonication for several minutes. After that, HA-AC was further completely dissolved in NanoDOX solution at a concentration of 80 mg mL^−1^. Then, MMP cross-linker was dissolved in the same volume of TEOA buffer at a concentration of 20 mg mL^−1^. After mixing NanoDOX/HA-AC solution and MMP cross-linker solution through a disposal-connected mixing system, NDHM was completely formed within 5 min.

### 2.8 *In vitro* DOX release from NDHM

The *in vitro* DOX release from NDHM was studied in fresh PBS (150 mM, pH = 7.4) with or without MMP-2 (1.5 μg mL^−1^). A 0.15 mL volume of NDHM in 2 mL of PBS (150 mM, pH = 7.4) with or without MMP-2 was placed in a dialysis bag with a MWCO of 3500 Da. At each specified time point, 2 mL of the release medium was withdrawn from the bottles and replaced with 2 mL of fresh PBS (150 mM, pH = 7.4). The released DOX concentrations in the 2 mL solutions were measured by an UV-vis spectrophotometer at 500 nm. The cumulative drug release was calculated by the following formula:
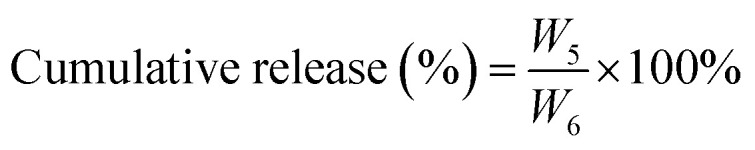
where *W*_5_ is the weight of DOX cumulatively released from gels at a specified time point and *W*_6_ is the weight of DOX loaded into the gels.

### 2.9 Animal model

BALB/c nude mice (4–6 weeks old) were purchased from Vital River Laboratory Animal Technology Co. Ltd. (Beijing, China). All animal procedures were performed in accordance with the Guidance Suggestions for the Care and Use of Laboratory Animals and approved by the Institutional Animal Care and Use Committee of Beijing Vital River Laboratory Animal Technology Co. Ltd.

To make mice tumor-bearing, BALB/c nude mice were subcutaneously injected (right upper back) with SCC-15 cells (5 × 10^6^ cells per mouse) suspended in 200 μL of the mixture of serum-free DMEM and high-protein Matrigel (BD, CA, USA) (volume ratio = 1 : 1). The tumor size was measured twice weekly with a caliper-like instrument in two dimensions. The tumor volume was calculated using the following formula:
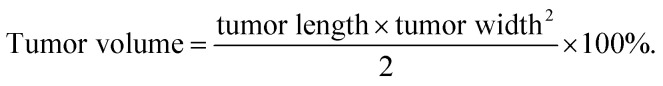


### 2.10 *In vivo* hydrogel degradation test

To test the *in vivo* degradability of the hydrogel, normal mice and tumor-bearing mice were prepared for the degradation of the hydrogels. The mice were subcutaneously injected (right upper back) with 100 μL of blank hydrogel. At different appointed times (1 d, 7 d, and 14 d), the mice were euthanized and dissected to expose the injection site, which would be observed and photographed.

### 2.11 *In vitro* cellular uptake

SCC-15 cells were seeded in 8-well LabTek chambered cover glass systems (8 × 10^4^ cells per well) in 0.2 mL of Dulbecco's modified Eagle's medium (DMEM, HyClone, Utah, USA) supplemented with 10% fetal bovine serum (FBS, Gibco, MA, USA), 100 units per mL of penicillin, and 100 μg mL^−1^ streptomycin. The cells were allowed to attach and grow overnight at 37 °C in 5% CO_2_. The cells were then incubated with 0.4 mL of growth medium and 80 μL of normal saline (NS), free DOX, NanoDOX and NDHM at 37 °C for 24 h. The cells were washed with PBS (HyClone, Utah, USA) and then imaged using confocal laser scanning microscopy (CLSM, TCSSP2, Leica, Germany).

### 2.12 *In vitro* MTT assay

SCC-15 cells were planted into 96-well plates (4000 cells per well) and incubated in a carbon dioxide incubator at 37 °C in 5% CO_2_ overnight. After that, the gel precursor solution was then placed between two Teflon plates for 30 min at 37 °C to allow for gelation, and then, the pieces of gel with final DOX concentrations of 0 to 160 μg mL^−1^ were put in the well to be incubated with the cells for 0.5 d, 1 d, 2 d or 4 d. After incubation, 20 μL of sterile 3-[4,5-dimethylthiazol-2-yl]-2,5-diphenyltetrazolium bromide (MTT) stock solution (5 mg mL^−1^ in PBS) was added to each well. After 4 h, the drug-loaded hydrogel and the cell culture medium were discarded, and the excess MTT was washed with PBS buffer. The formazan crystals were dissolved in dimethyl sulfoxide (100 μL per well), and the absorbance was measured with a microplate reader (Multiskan MK3, Thermo Scientific) at a wavelength of 570 nm. The cell viability (%) was calculated by this equation:
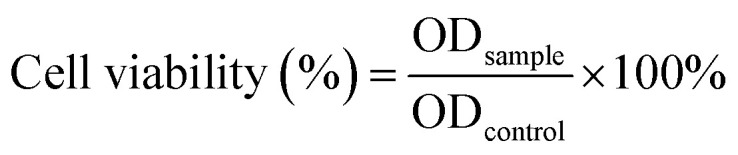
where the sample is the cells treated with drug-loaded hydrogel and the control is the cells treated with NS.

### 2.13 *In vitro* live-dead assay

The antitumor efficacy was further studied through live/dead staining. First, SCC-15 cells were seeded in 8-well LabTek Chambered Cover glass systems at a density of 7000 cells per well and cultured in a carbon dioxide incubator at 37 °C overnight. Then, the hydrogels with a final DOX concentration of 40 μg mL^−1^ were added into wells to be cultured with cells for 24 h. After incubation, the gels and DMEM were discarded. The cells were washed three times with PBS and further stained with a live/dead viability/cytotoxicity kit (Molecular Probes, Eugene, OR) using the manufacturer's protocol. After staining for 30 min at 37 °C in the dark, the cells were imaged with a CLSM.

### 2.14 *In vitro* apoptosis assay

Cell apoptosis, an active process of gene regulation, was also studied by using an annexin-V/PI assay on a CLSM. After antitumor treatment that was the same as for the *in vitro* live-dead study, the cells were examined by an Annexin V-FITC Apoptosis Detection kit (BD Biosciences, CA, USA). Cells were stained by annexin V-FITC and PI and then washed with PBS, followed by removal of the medium and imaging with a CLSM.

### 2.15 *In vitro* wound healing assay

Cells (2 × 10^5^) were seeded on 6-well plates. After being cultured overnight at 37 °C in 5% CO_2_, some artificial “wounds” were carefully created on the surface by using a 20 μL pipette tip to scratch the confluent cell layer. Cells were washed twice to remove detached cells and debris. After antitumor treatment that was the same as for the *in vitro* live-dead study, observation of the level of cell migration was started at 0 h as the control and carried out for a specified time using an inverted microscope.

### 2.16 *In vivo* fluorescence imaging

When the tumors were approximately 200 mm^3^ in volume, the mice were randomly divided into four groups (*n* = 3) and treated locally with NS, free DOX (DOX·HCl, the dose of DOX at 5 mg kg^−1^) and NDHM (the dose of DOX at 5 mg kg^−1^), respectively. Their distributions were imaged using IVIS after 0 h, 1 h, 12 h, 24 h, 2 d, 3 d and 5 d. All of the mice were euthanized after 5 d, and their organs (brain, heart, liver, spleen, lungs, and kidneys) and tumors were collected for imaging. The fluorescence intensity was measured using IVIS software.

### 2.17 *In vivo* tumor growth inhibition

To evaluate the efficacy of NDHM *in vivo*, SCC-15 tumor-bearing mice were randomly divided into four groups with five mice in each group. When the tumors reached 100 mm^3^ in volume, the mice were treated with NS, free DOX (DOX·HCl, the dose of DOX at 5 mg kg^−1^), NDHM-1 (the dose of DOX at 2.5 mg kg^−1^) or NDHM-2 (the dose of DOX at 5 mg kg^−1^) *via* local delivery at 0 d and 17 d. The tumor volumes and the general condition of the mice were recorded twice weekly. After 35 d, the mice were euthanized, and the excised tumors were weighed.

The histopathological changes in the tumor tissue were confirmed by preparation and staining (hematoxylin-eosin (H&E), terminal deoxynucleotidyl transferase-mediated dUTP nick end labeling (TUNEL)) of the tumor tissue. The excised tumors were fixed in formalin, and then, they were embedded in paraffin and sectioned into slices with a thickness of 4 μm. After that, all of the sections were further stained with H&E and TUNEL. The H&E sections and the TUNEL sections were observed by a CLSM. To systematically study the effects of NDHM on the whole body of nude mice, the main excised organs (brain, heart, liver, spleen, lungs, and kidneys) were fixed in formalin, and then, they were embedded in paraffin and sectioned into slices with a thickness of 4 μm. After that, all of the sections were further stained with H&E and observed by CLSM.

### 2.18 Statistical analysis

The results were expressed as the mean ± SD. All statistical analyses were performed using GraphPad (InStat, CA, USA). Significant intergroup differences were determined using one-way ANOVA followed by Tukey's post-test; *P* < 0.05 was considered significant.

## 3 Results and discussion

### 3.1 Synthesis and characterization of polymer materials

Amphiphilic PDLLA-PEG-PDLLA triblock copolymers were synthesized by the ring-opening polymerization of d,l-lactide using PEG as an initiator and stannous octoate as a catalyst (Scheme S1†). ^1^H NMR and GPC analyses were used to characterize the chemical structure of PDLLA-PEG-PDLLA copolymers. As shown in Fig. S1,† the proton peak at 3.7 ppm is attributed to methylene of the PEG block, and the peaks at 5.2 ppm and 1.8 ppm belong to methine and methyl of the PDLLA block.^40^ Furthermore, the GPC result shows that the *M*_w_ of the copolymers is 18 168 Da (Fig. S2†). All of these results indicated that PDLLA-PEG-PDLLA triblock copolymers were successfully synthesized.

HA-AC was synthesized through a two-step modification with ADH and NAS (Scheme S2†). ^1^H NMR analyses of the products of each step were performed and are shown in Fig. S3.† Compared to the spectrum of raw HA, the new proton peaks of methylene and vinyl appeared at 1.6 ppm, 2.3 ppm, and 6.2 ppm, indicating that ADH and NAS were successfully modified on HA and HA-AC was obtained.^43^

### 3.2 Preparation and characterization of NanoDOX

**Fig. 1:**
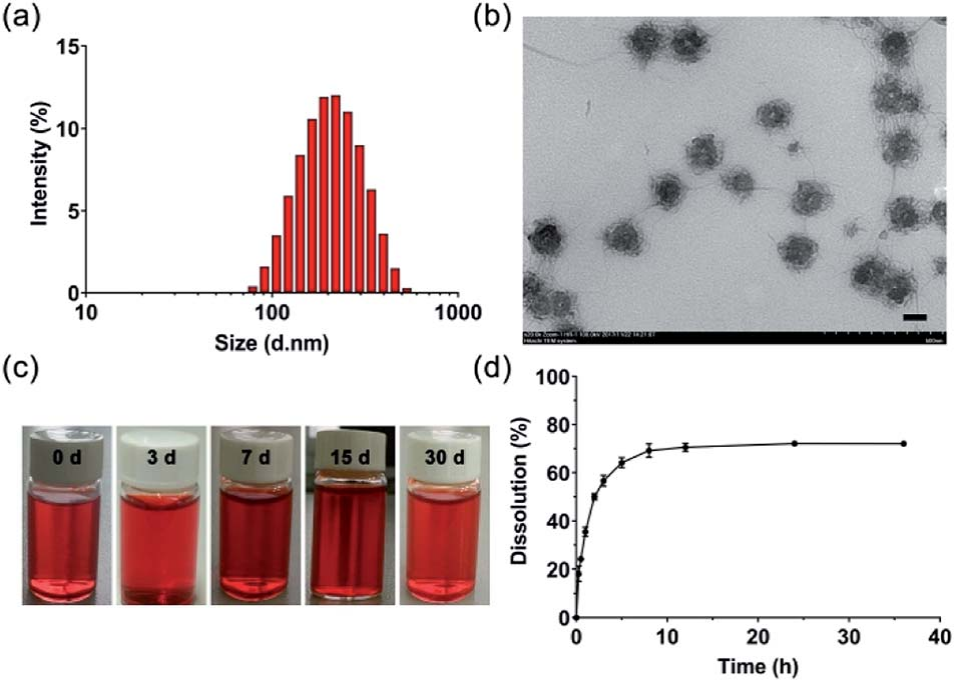
(a) Size distribution of NanoDOX; (b) TEM image of NanoDOX (bar = 100 nm); (c) photographs of NanoDOX dispersed in PBS (pH = 7.4) at 1, 3, 7, 15 and 30 d; and (d) cumulative release of DOX from NanoDOX dispersed in PBS at 37 °C (pH = 7.4). Data represent means ± SD (*n* = 3).

DOX-loaded PDLLA-PEG-PDLLA micelles (NanoDOX) were prepared using a solvent-antisolvent process.^44^ The drug-loading capacity and the encapsulation efficiency of NanoDOX were approximately 70.1% and 3.5%, respectively, confirming that DOX was successfully encapsulated in PDLLA-PEG-PDLLA micelles. Fig. 1a and S4† show the size and zeta potential of NanoDOX. An average diameter of 220.8 nm and zeta potential of −11.4 mV were observed. Furthermore, TEM images exhibited that NanoDOX had a spherical shape with a uniform particle size of approximately 150–200 nm (Fig. 1b), which was consistent with the DLS results. In particular, the internal structure of NanoDOX presented a dark DOX core and gray polymer layer built by PDLLA-PEG-PDLLA. To verify the aqueous dispersion and stability, NanoDOX was dispersed in PBS buffer for a long incubation time. As shown in Fig. 1c, the NanoDOX solution was highly transparent and no sediment appearing event occurred after 30 d. Fig. S5† shows the changes of particle size of NanoDOX during 30 d incubation by DLS test. The average particle size was slightly increased from 220 nm to about 340 nm, indicating that NanoDOX had better dispersibility and stability in the aqueous phase. The drug release profile of NanoDOX is shown in Fig. 1d. Approximately 70% of loaded DOX was released during the initial 10 h in PBS buffer at 37 °C, suggesting that NanoDOX had an outstanding drug release profile during incubation in the biological environment.

### 3.3 Preparation and characterization of NDHM

**Fig. 2:**
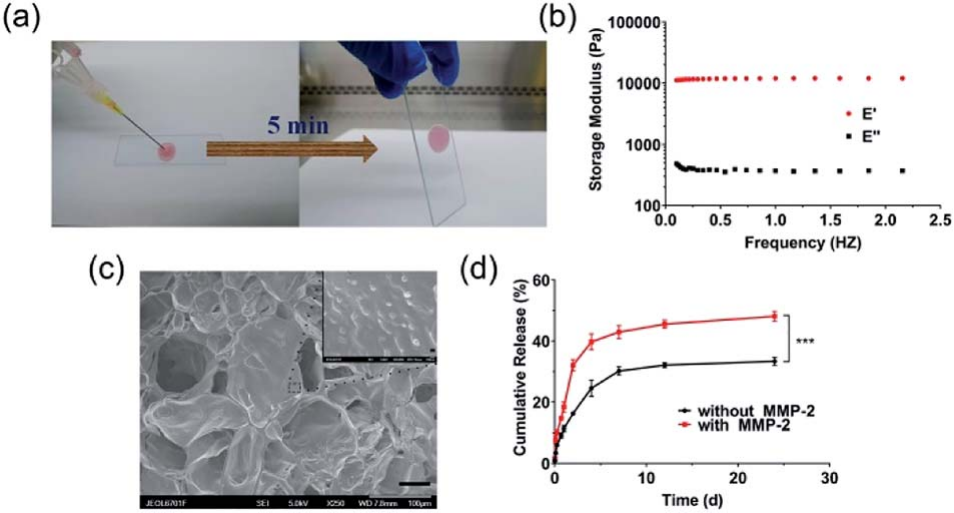
(a) Gelling process: NanoDOX/HA-AC solution and MMP cross-linker solution were mixed by a disposal-connected mixing system, and the mixture was solidified after 5 min of injection; (b) DMTA of NDHM in the frequency range of 0.1 Hz to 2 Hz at 37 °C; (c) SEM image of lyophilized NDHM (scale bar = 50 μm); and (d) cumulative release of DOX from NDHM with or without MMP-2. Data represent the mean ± SD (*n* = 3). ***P < 0.001.

NDHM was prepared by mixing a HA-AC solution containing NanoDOX with a MMP cross-linker solution through a disposal-connected mixing system. As shown in Fig. 2a, the hydrogel precursor solution was completely solidified after 5 min of injection, suggesting that the formation time of the hydrogel will be quick enough for *in vivo* applications.

The mechanical properties of the hydrogel are shown in Fig. 2b. For the hydrogels, *E*′ was consistently higher than *E*′′, and they exhibited a plateau over the entire frequency range, indicating that the hydrogels were robust and behaved as elastic solids.^45,46^

SEM imaging was performed to examine the distribution of NanoDOX in the hydrogels. As displayed in Fig. 2c, NanoDOX was found to be sticking on hydrogel matrices as monodisperse particles, which would be beneficial for achieving a long-term sustained DOX release based on both the molecular diffusion and the degradation of the hydrogel host material.

To evaluate the MMP-2-mediated drug release features, NDHM were incubated in the presence and absence of MMP-2 in a pH of 7.4 buffer at 37 °C. As shown in Fig. 2d, the DOX release rate of the group in the presence of MMP-2 was noticeably faster than that of the group in the absence of MMP-2, demonstrating that the DOX released from the hydrogel was supplied not only by spontaneous molecule diffusion but also by the degradation of hydrogel matrices that was caused by the breaking of MMP cross-linker in the presence of MMP-2. In particular, the loading of NanoDOX into hydrogels significantly extended the drug release time, and approximately 30% and 50% of DOX was released from the hydrogels in the presence and absence of MMP-2 during 24 d of incubation, respectively, indicating that this NDHM drug delivery system has an outstanding long-term sustained drug release property.

### 3.4 *In vivo* degradation investigation of HA-MMP hydrogels

**Fig. 3:**
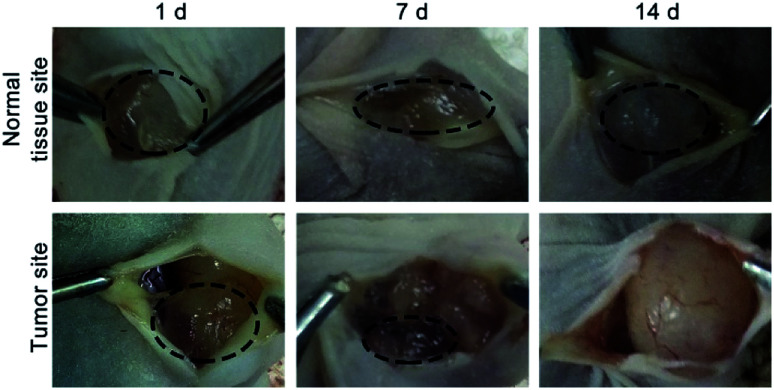
Representative images of the remaining hydrogels under the skin of the normal tissue site or tumor site of nude mice at 1 d, 7 d and 14 d.

To investigate the MMP-responsive degradability of HA-MMP hydrogels *in vivo*, the hydrogel precursor solution was injected into normal tissue and the tumor site of SCC-15 tumor-bearing mice, and the mice were dissected at 1, 7 and 14 d. Compared to normal tissue, the hydrogels in the tumor site showed a more rapid degradation rate, and almost all of the gels were degraded at 14 d (Fig. 3), suggesting that MMP-2 overexpressed in the tumor site accelerated the degradation of HA-MMP hydrogels.

### 3.5 *In vitro* cellular uptake and cell cytotoxicity of NDHM

**Fig. 4:**
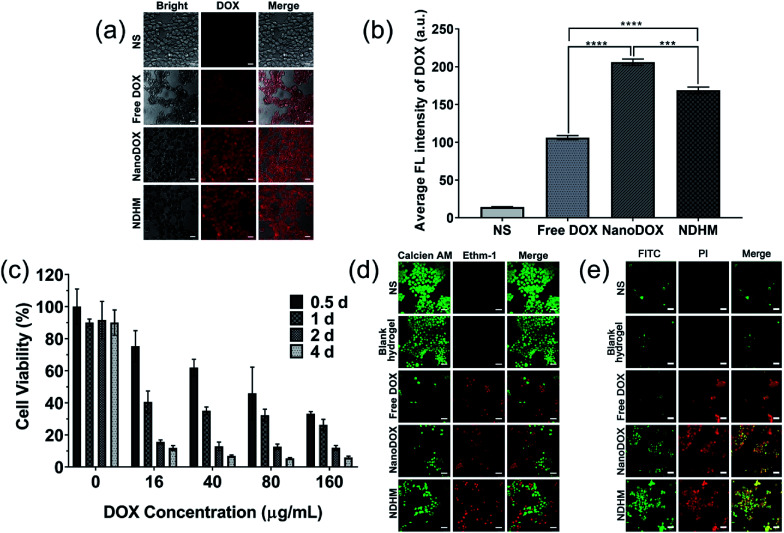
(a) CLSM images showing the cellular distribution of DOX (red) in SCC-15 cells that were treated with NS, free DOX, NanoDOX, and NDHM at a 16 μg mL^−1^ DOX concentration in the medium (scale bar = 25 μm); (b) the fluorescence quantitative analysis of the cellular uptake of DOX. Data represent means ± SD (*n* = 3), ***P < 0.001, ****P < 0.0001. (c) Relative cell viabilities of SCC-15 cells after treatments with different concentrations of DOX in HA-MMP hydrogels for 0.5 d, 1 d, 2 d, and 4 d. Data represent means ± SD (*n* = 3). (d) CLSM images of SCC-15 cells stained with calcein AM and Ethm-1 upon different treatments with NS, blank hydrogel, free DOX, NanoDOX, and NDHM at a 16 μg mL^−1^ DOX concentration in the medium (scale bar = 50 μm). (e) CLSM images of SCC-15 cells stained with FITC and PI upon different treatments with NS, blank hydrogel, free DOX, NanoDOX or NDHM at a 16 μg mL^−1^ DOX concentration in the release medium (scale bar = 50 μm).

CLSM was used to monitor the uptake of DOX by the SCC-15 cancer cells.^47^ As shown in Fig. 4a and b, there was strong red fluorescence of DOX observed in the cells treated with NanoDOX. Furthermore, after incubation with NDHM, the cells also presented red DOX fluorescence, explaining that the hydrogels can release the loaded NanoDOX for cellular uptake.

The cytotoxicities of blank hydrogels and NDHM with various DOX concentrations were evaluated at different treatment times (0.5 d, 1 d, 2 d and 4 d) by the MTT assay. As shown in Fig. 4c, HA-MMP hydrogels presented excellent biocompatibility, with more than 90% of cells remaining alive even after treatment for 4 d. The MTT assay of NDHM exhibited both drug concentration- and treatment time-related cytotoxicity. At the highest drug concentration of 160 μg mL^−1^ and treatment period of 4 d, the cell survival rate was only approximately 5.9%.

To visually observe live and dead cells, the cells were incubated with various samples for 24 h, and the live-dead assay method was adopted. Fig. 4d shows that upon treatment with either NS or blank hydrogel, nearly all of the cells presented green fluorescence, demonstrating their good survival. After treatment with free DOX, NanoDOX and NDHM, dead cells with red fluorescence appeared. Although NDHM exhibited relatively low cytotoxicity compared with free DOX and NanoDOX, it still can cause a large number of cell deaths during 24 h of treatment.

Annexin-V/PI double-staining was used for labeling the apoptotic or necrotic cells treated by various samples for 24 h, and the results were detected by a CLSM. As shown in Fig. 4e, after treatment with either NS or blank hydrogel, there was no obvious sign of apoptotic or necrotic cells. Furthermore, the cells treated with free DOX and NanoDOX exhibited that almost all of them remained late apoptotic/necrotic by the red fluorescence presented. The cells incubated with NDHM showed more green fluorescence than the cells incubated with either free DOX or NanoDOX, indicating that NDHM had a sustained drug release behavior and could induce cell-programmed apoptosis.

### 3.6 *In vitro* wound healing assay of NDHM

**Fig. 5:**
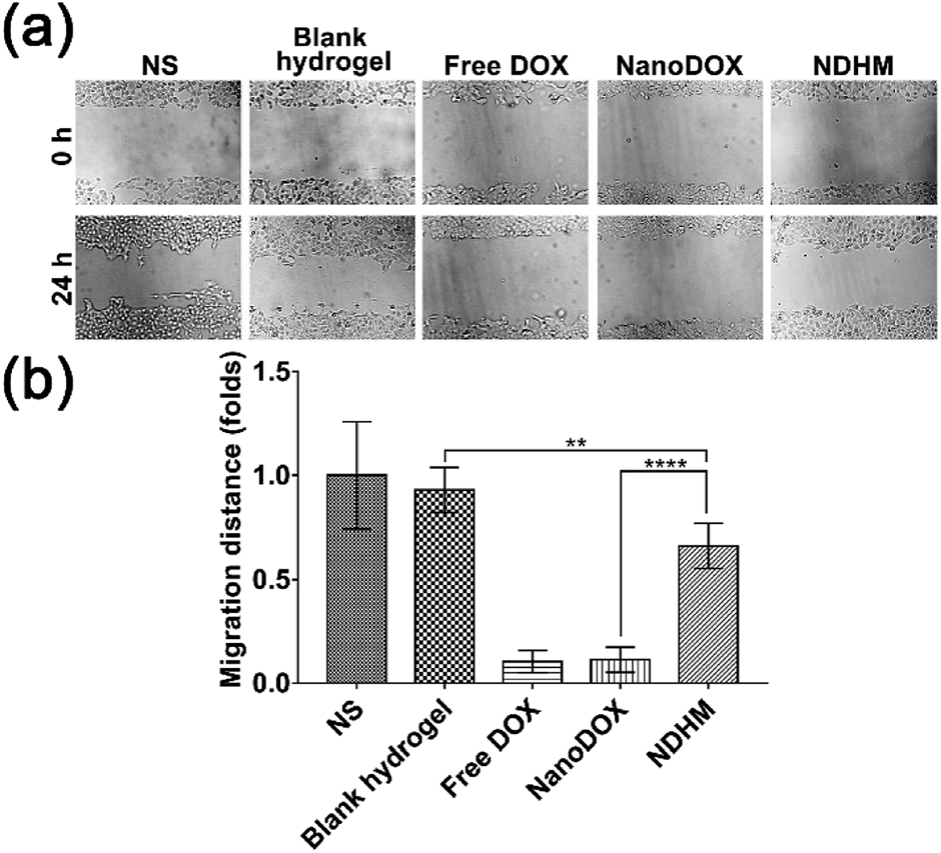
(a) Wound healing assay of SCC-15 cells treated with NS, blank hydrogel, free DOX, NanoDOX, and NDHM at a 16 μg mL^−1^ DOX concentration. The cells were imaged by an inverted microscope at 0 h and 24 h. (b) The quantitative analysis of the wound healing assay. Data represent means ± SD (*n* = 5), **P < 0.01, ****P < 0.0001.

To investigate the effects of NS, blank hydrogel, free DOX, NanoDOX and NDHM on cell migration, we performed a scratch-wound healing assay to detect the cell movement behavior. As shown in Fig. 5a and b, the scratched areas in the blank hydrogel treatment group had significantly narrowed and even performed no differently from the NS group at 24 h. For the hydrogel loaded with NanoDOX, the inhibition of cell migration was not as serious as that of free DOX or NanoDOX but more obvious than that of the blank hydrogel.

### 3.7 *In vivo* fluorescence imaging

**Fig. 6:**
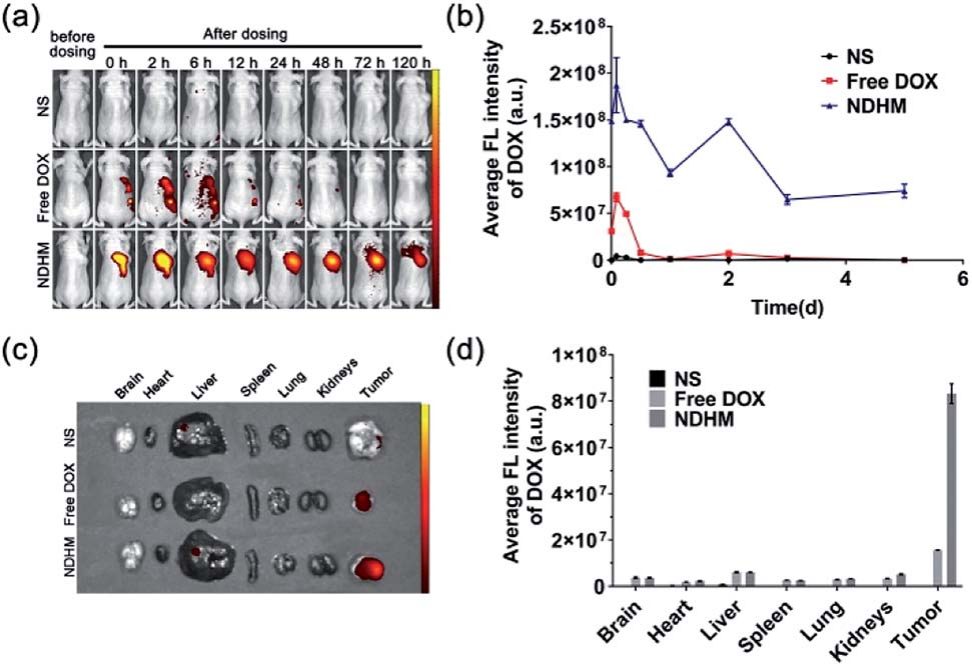
(a) *In vivo* fluorescence imaging of mice bearing SCC-15 tumors at different time points after the *in situ* injection of NS, free DOX, and NDHM (the dose of DOX at 5 mg kg^−1^); (b) average fluorescence intensity of the tumor site by quantitative analysis. Data represent means ± SD (*n* = 3); (c) *ex vivo* fluorescence imaging of the tumor and main organs at 5 d; and (d) average fluorescence intensity of the tumor and main organs by quantitative analysis. Data represent means ± SD (*n* = 3).

To validate that HA-MMP hydrogels can remain at the tumor site for local and sustained drug release, free DOX and NDHM were locally injected into the tumor site of the BALB/c nude tumor-bearing mice, and fluorescent images were subsequently recorded at different time intervals by an *in*/*ex vivo* imaging system. As displayed in Fig. 6a and b, for the injection of the free DOX group, the DOX fluorescence signal was substantially reduced from 6 h to 12 h, and it almost completely disappeared 24 h after administration. In contrast, the mice injected with NDHM exhibited a higher fluorescence intensity for every studied time interval, and even at 5 d, the post-administration fluorescence signal still remained over 50% compared to the initial state (Fig. 6b). In particular, the fluorescence signal of NDHM was mainly located at the tumor site during the first 3 d and slightly spread to the surrounding tissues at 5 d after administration. Additionally, the mice were sacrificed after 5 d post-administration, and various organs and the tumor were isolated for *ex vivo* imaging for further investigation of the biodistribution of the fluorescence signal of DOX. As displayed in Fig. 6c, no fluorescence signal of DOX appeared in the liver, spleen, lungs, and kidneys for both free DOX and NDHM, and only the tumor presented a brighter fluorescence signal. Furthermore, as shown in Fig. 6d, the average fluorescence intensity of DOX in the tumor tissue from the NDHM group was 5.3-fold higher than that from the free DOX group. Taken together, these *in*/*ex vivo* imaging results verified that HA-MMP hydrogels are able to hold NanoDOX at the tumor site for sustained and/or stimuli-responsive drug release over a long-term period.

### 3.8 *In vivo* tumor growth inhibition

**Fig. 7:**
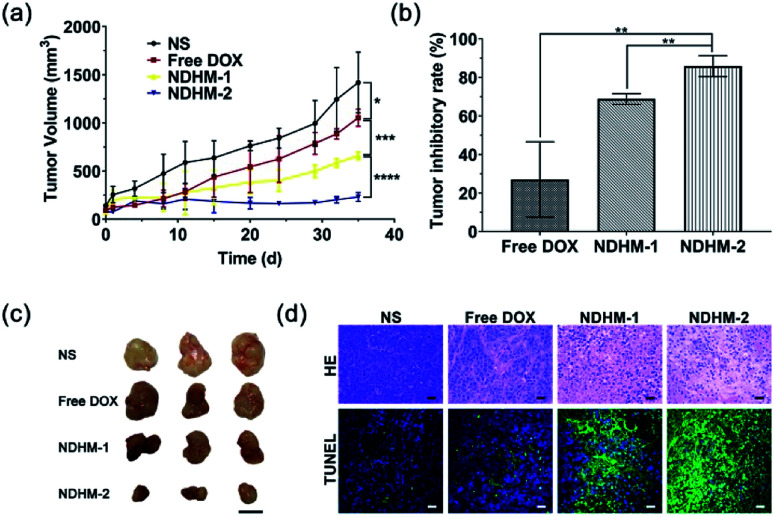
(a) Growth curves of SCC-15 tumors in nude mice after treatments with NS, free DOX, NDHM-1 (the dose of DOX at 2.5 mg kg^−1^) and NDHM-2 (the dose of DOX at 5 mg kg^−1^). Data represent means ± SD (*n* = 6), *P < 0.1, ***P < 0.001, ****P < 0.0001; (b) the tumor inhibitory rate after treatment. Data represent means ± SD (*n* = 6), **P < 0.01; (c) representative mice photographs of each group were recorded before and after treatments; and (d) hematoxylin and eosin (H&E) staining and TdT-mediated dUTP nick-end labeling (TUNEL) staining of the tumor tissues after different treatments (scale bar = 25 μm).

To assess the *in vivo* tumor inhibition efficacy, free DOX and HA-MMP hydrogels loading with different doses of NanoDOX (NDHM-1 and NDHM-2) were locally injected into the tumor site of the BALB/c nude tumor-bearing mice for two treatments at 0 and 17 d. As shown in Fig. 7a, compared to the saline group, tumor growth was significantly inhibited after injecting free DOX, NDHM-1 and NDHM-2 during the first 11 d, and after that, the tumor treated with free DOX started to grow in a fast manner. However, the mice treated with NDHM-1 and NDHM-2 exhibited a sustained and outstanding effect of tumor growth inhibition, and the inhibition rates were 68.7% and 85.9% at 35 d for the NDHM-1 and NDHM-2 groups, respectively (Fig. 7b and c). To further evaluate the *in vivo* antitumor effect, the histological analysis of tumors is shown in Fig. 7d. Compared to the NS and free DOX groups, both the NDHM-1 and NDHM-2 groups exhibited more obvious nuclear condensation and fragmentation in the H&E images, demonstrating that NDHM-1 and NDHM-2 had a better antitumor activity. Moreover, the tumors treated with NDHM-1 and NDHM-2 presented the highest level of cell apoptosis, as indicated by the more green fluorescent cells that appeared in the tumor section stained by the TUNEL assay, suggesting that the outstanding capability of tumor growth inhibition was partly due to the promotion of apoptosis induced by NDHM (Fig. 7d). Overall, it was clarified that NDHM had prominent and long-term tumor inhibition efficacy.

### 3.9 Biosafety evaluation

The *in vivo* toxicity of NDHM was evaluated in term of the body weight change and histological analysis of the main organs. All of the mice were alive during the treatment. In addition, compared with mice treated with NS, there was no significant loss in the body weight after the treatments with NDHM-1 and NDHM-2 (Fig. S6†). Furthermore, histological analysis of H&E staining demonstrated that the groups of mice treated with both NDHM-1 and NDHM-2 had no noticeable histopathological change in any of the tested organs, as shown in Fig. S7.† Moreover, all of the results confirmed that NDHM had no evident toxicity to nude mice, suggesting the security for clinical application prospects.

## 4 Conclusions

We successfully developed a local chemotherapy drug delivery system based on PDLLA-PEG-PDLLA copolymer micelles and an *in situ* forming injectable HA-MMP hydrogel. *In vitro* dissolution studies showed that drug-loaded hydrogels had excellent sustained and MMP-2-responsive drug release profiles. The cytotoxicity investigations demonstrated that drug-loaded hydrogels could induce programmed apoptosis and exhibited high toxicity for the SCC-15 cell-line. Furthermore, the *in vivo* study proved that drug-loaded hydrogels were located at tumor sites for a long-term inhibitory effect on tumor growth with outstanding biosafety features in xenograft models of squamous cell carcinoma in nude mice. Moreover, we believe that our work presented a promising local stimuli-responsive drug delivery system, which is not limited to use for the treatment of solid tumors and can be adapted for the prevention of the postoperative recurrence of tumors, and the relative studies are in progress.

## Conflicts of interest

There are no conflicts to declare.

## Supplementary Material

RA-009-C9RA04343H-s001
