# Supplementary material for: MMP-responsive in situ forming hydrogel loaded with doxorubicin-encapsulated biodegradable micelles for local chemotherapy of oral squamous cell carcinoma
Source: RSC Adv. 2019 Oct 2;9(54):31264–73. doi: 10.1039/c9ra04343h (PMC9072589; doi:10.1039/c9ra04343h)
Supplement: RA-009-C9RA04343H-s001 [file RA-009-C9RA04343H-s001.pdf]

Electronic Supplementary Material (ESI) for RSC Advances.
This journal is © The Royal Society of Chemistry 2019

Electronic Supplementary Information

MMP-responsive *in-situ* forming hydrogel loaded with doxorubicin-encapsulated biodegradable micelles for local chemotherapy of oral squamous cell carcinoma

Wei Li,[a] Cheng Tao,[a] Jiexin Wang,*[abc] Yuan Le,[ac] Jianjun Zhang*[a]

[a]State Key Laboratory of Organic-Inorganic Composites, College of Chemical Engineering, Beijing University of Chemical Technology, Beijing 100029, PR China.

[b]Beijing Advanced Innovation Center for Soft Matter Science and Engineering, Beijing University of Chemical Technology, Beijing, 100029, PR China.

[c]Research Center of the Ministry of Education for High Gravity Engineering and Technology, Beijing University of Chemical Technology, Beijing, 100029, PR China.

E-mail: zhangjj@mail.buct.edu.cn; wangjx@mail.buct.edu.cn.

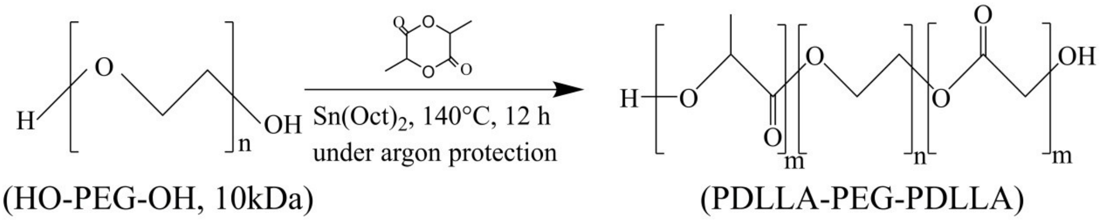

Scheme S1 Synthetic route of poly(D,L-lactide)-poly(ethylene glycol)-poly(D,L-lactide) (PDLLA-PEG-PDLLA).

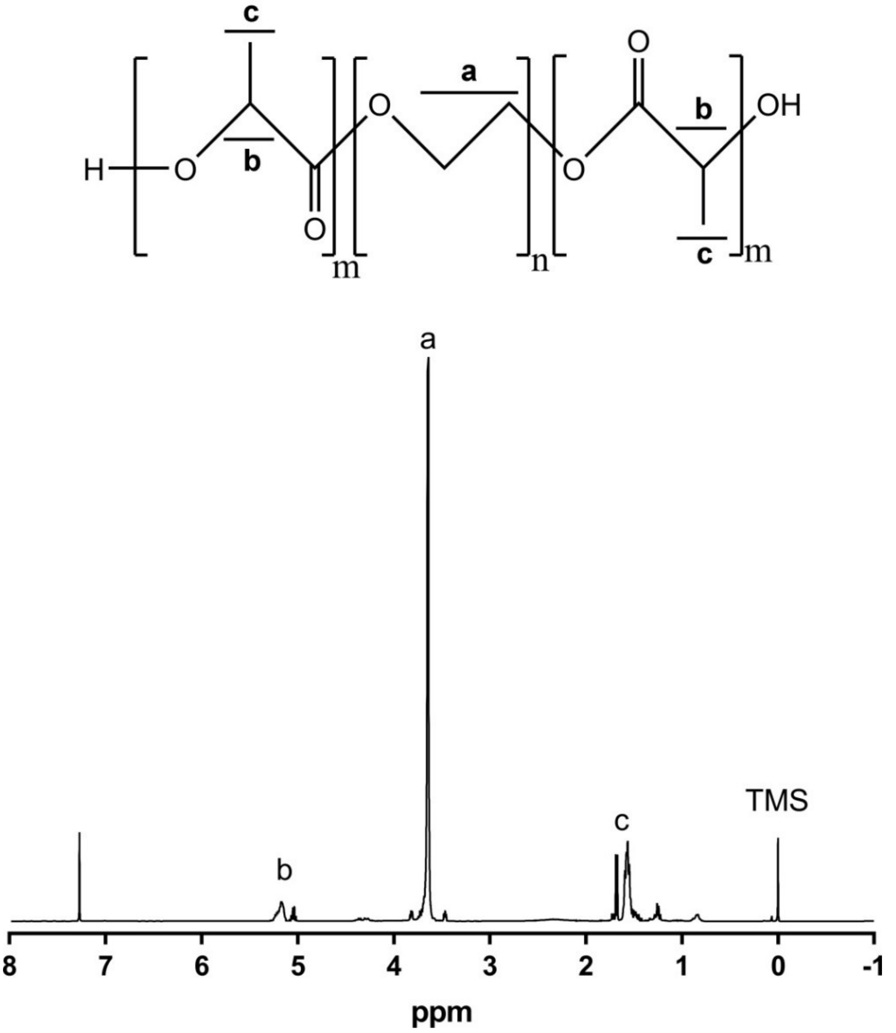

Fig. S1 ^{1}H NMR spectra of PDLLA-PEG-PDLLA using $CDCl_3$ as solvent. δ=3.7 ppm (PEG chain protons), 5.2 ppm (methine of PDLLA block) , 1.8 ppm (methyl of PDLLA block).

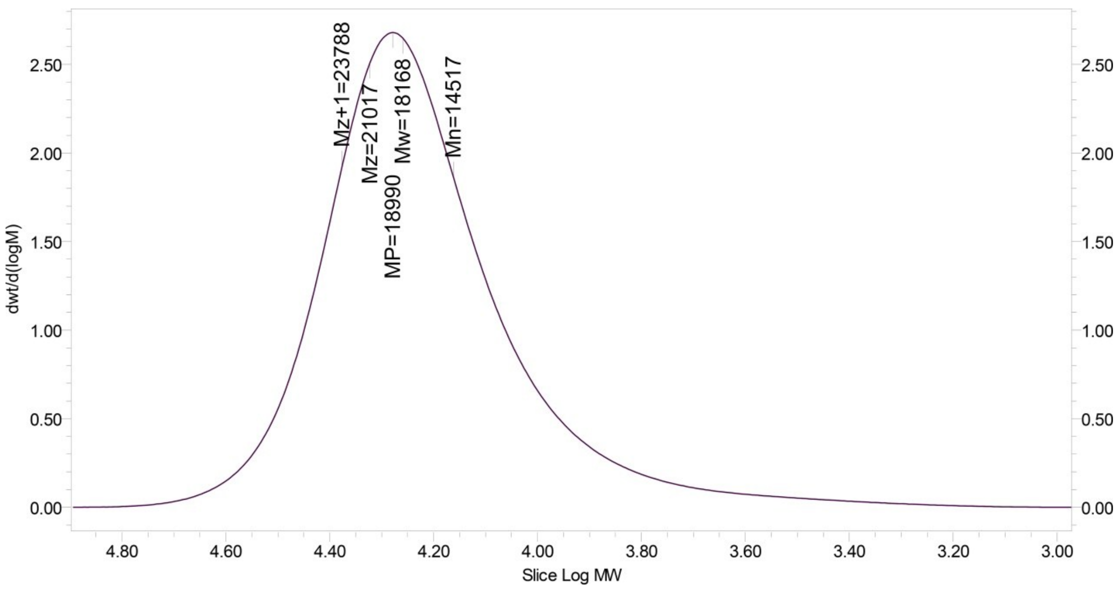

Fig. S2 Integral diagram of molecular weight of GPC of PDLLA-PEG-PDLLA.

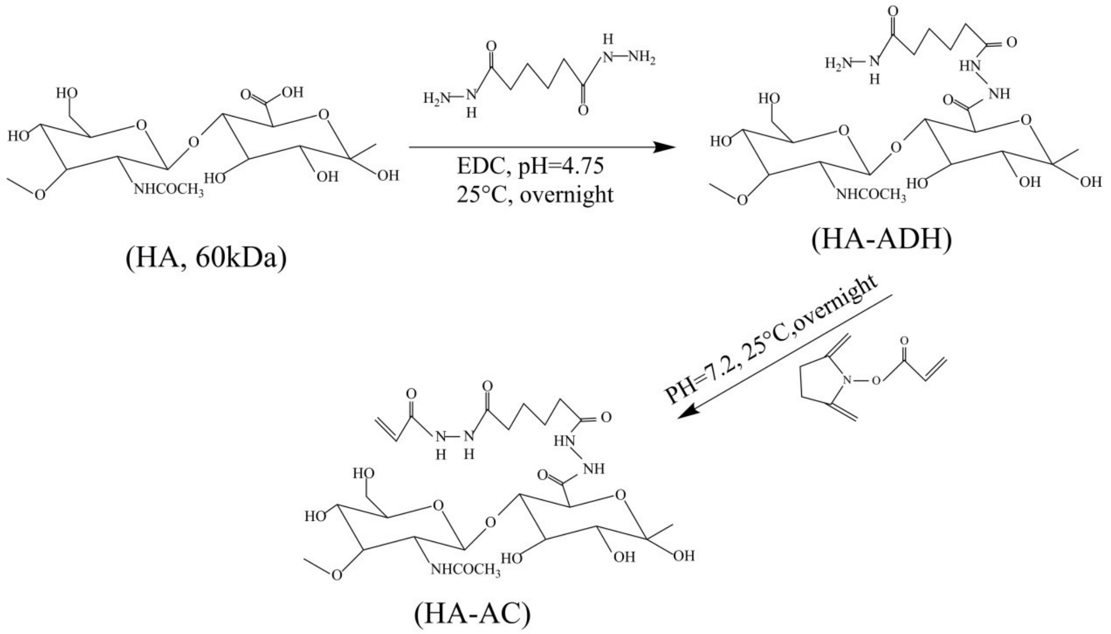

Scheme S2 Synthetic route of acrylated-hyaluronic acid (HA-AC).

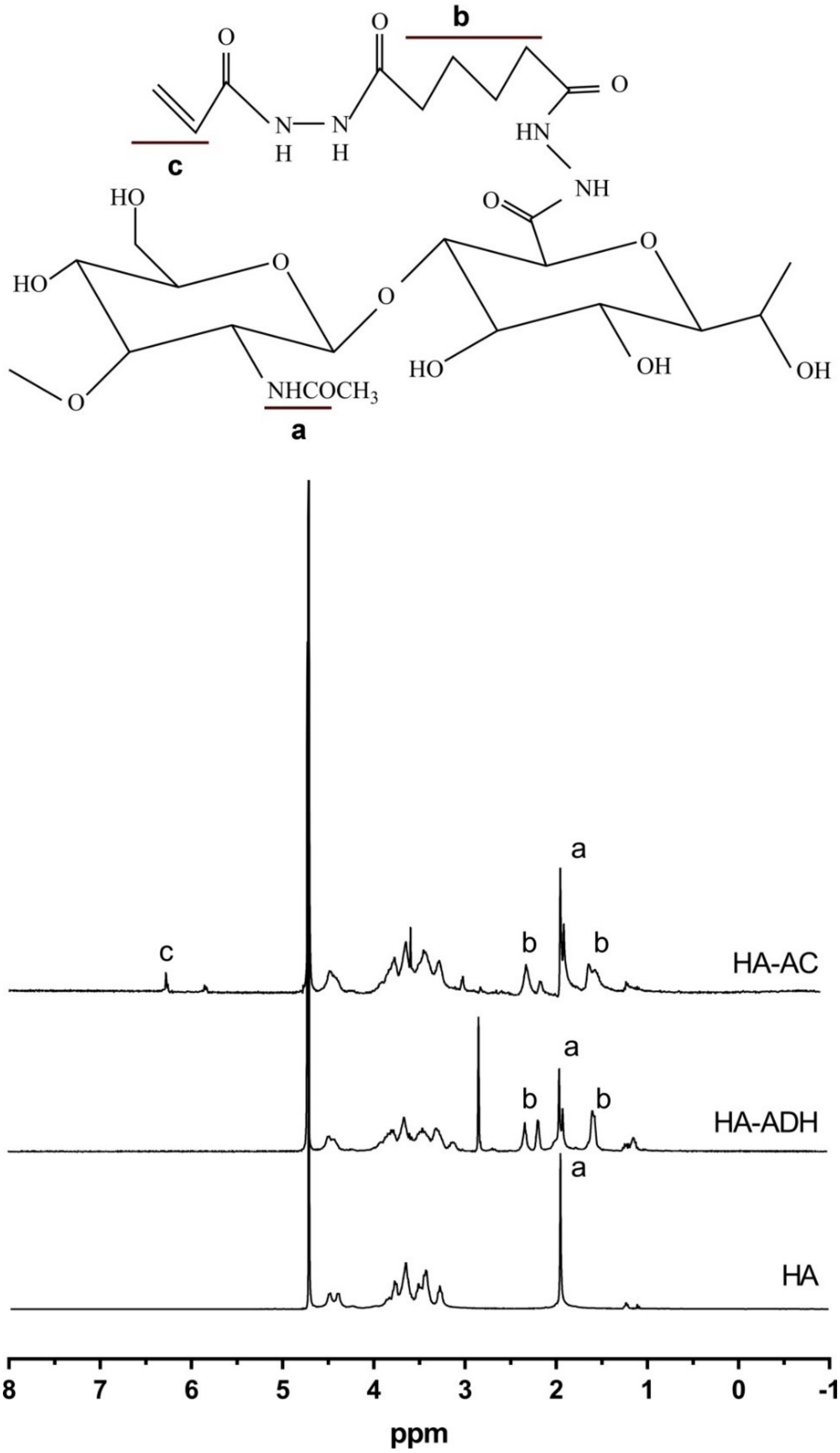

Fig. S3 ^{1}H-NMR spectrum of HA, HA-ADH and HA-AC. δ=1.6 ppm and 2.3 ppm (the eight hydrogens of the methylene groups on the ADH), 1.88 ppm (the acetyl methyl protons in HA), 6.2 ppm (the cis and trans acrylate hydrogens in HA-AC).

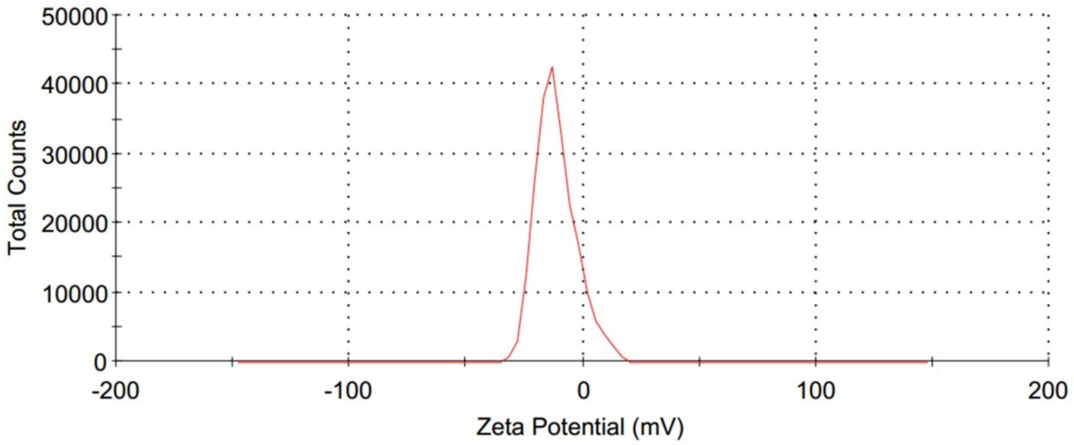

Fig. S4 Zeta potential distribution of NanoDOX in PBS (pH=7.4).

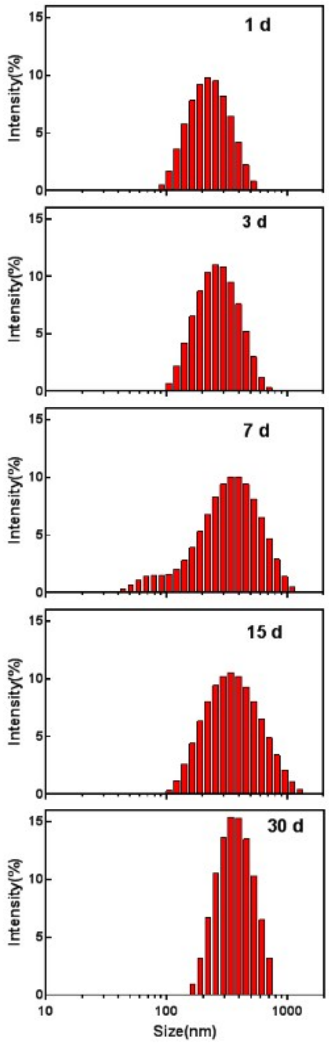

Fig. S5 The size distribution of NanoDOX incubated in PBS for 1, 3, 7, 15 and 30 d.

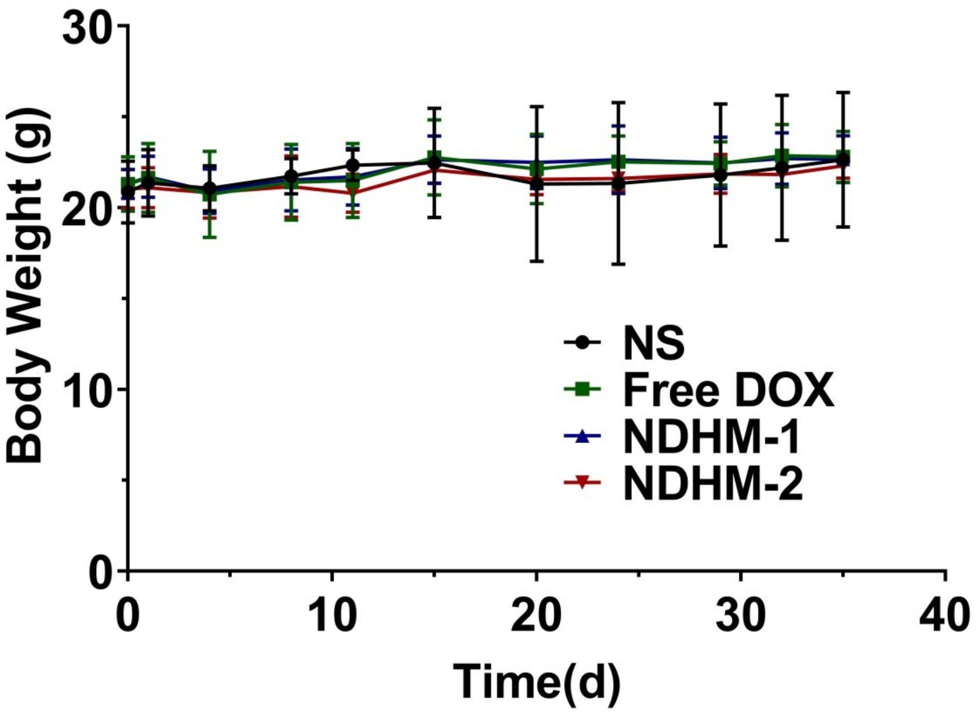

Fig. S6 Body weight curves of the mice for 35 days treated with NS, Free DOX, NDHM-1 (the dose of DOX at 2.5mg/kg) or NDHM-2 (the dose of DOX at 5mg/kg).

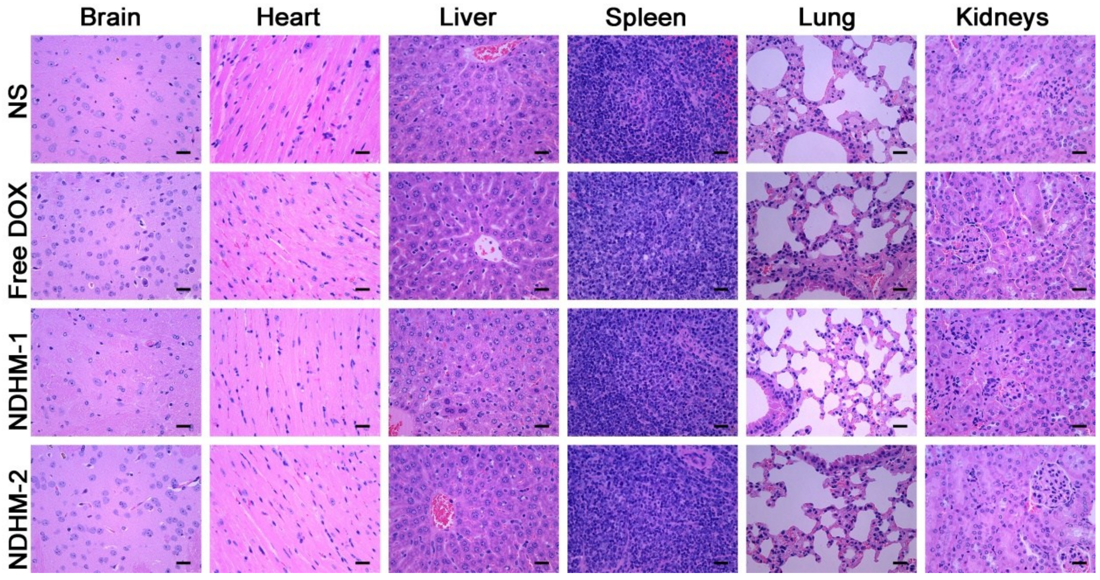

Fig. S7 Histological examination for mice treated with NS, free DOX, NDHM-1 (the dose of DOX at 2.5mg/kg) or NDHM-2 (the dose of DOX at 5mg/kg) (Scale bar=25μm).
